# Supplementary material for: Needle in a haystack? A comparison of eDNA metabarcoding and targeted qPCR for detection of the great crested newt (Triturus cristatus)
Source: Ecol Evol. 2018 May 29;8(12):6330–41. doi: 10.1002/ece3.4013 (PMC6024127; doi:10.1002/ece3.4013)
Supplement: Supplementary file 1 [file ECE3-8-6330-s001.pdf]

Supporting Information for:

Needle in a haystack? A comparison of eDNA metabarcoding and targeted qPCR for detection of the great crested newt (*Triturus cristatus*)

Lynsey R. Harper[1*], Lori Lawson Handley[1], Christoph Hahn[1,2], Neil Boonham[3,4], Helen C. Rees[5], Kevin C. Gough[6], Erin Lewis[3], Ian P. Adams[3], Peter Brotherton[7], Susanna Phillips[7] and Bernd Hänfling[1]

[1]*School of Environmental Sciences, University of Hull, Hull, HU6 7RX, UK*

[2]*Institute of Zoology, University of Graz, Graz, Styria, Austria*

[3]*Fera, Sand Hutton, York, YO14 1LZ, UK*

[4]*Newcastle University, Newcastle upon Tyne, NE1 7RU, UK*

[5]*ADAS, School of Veterinary Medicine and Science, The University of Nottingham, Sutton Bonington Campus, Leicestershire, LE12 5RD, UK*

[6]*School of Veterinary Medicine and Science, The University of Nottingham, Sutton Bonington Campus, Leicestershire, LE12 5RD, UK*

[7]*Natural England, Peterborough, PE1 1NG, UK*

***Corresponding author:** Lynsey Harper, L.Harper@2015.hull.ac.uk

Appendix 1: eDNA survey for great crested newt *Triturus cristatus*

SAMPLING

Twenty 30 mL water samples were collected at even intervals around the pond margin and pooled in a sterile 1 L Whirl-Pak® stand-up bag, which was shaken to provide a single homogenised sample from each pond. Six 15 mL subsamples were taken from the mixed sample using a sterile plastic pipette (25 mL) and added to sample tubes containing 33.5 mL absolute ethanol and 1.5 mL sodium acetate 3 M (pH 5.2) for ethanol precipitation. Subsamples were then sent to Fera (Natural England) and ADAS (private contracts) for eDNA analysis according to laboratory protocols established by Biggs *et al.* (2015). Subsamples were centrifuged at 14,000 x g for 30 minutes at 6 ^{o}C, and the supernatant discarded. Subsamples were then pooled during the first step of DNA extraction with the Qiagen® DNeasy Blood & Tissue Kit, where 360 µL of ATL buffer was added to the first tube, vortexed, and the supernatant transferred to the second tube. This process was repeated for all six tubes. The supernatant in the sixth tube, containing concentrated DNA from all six subsamples, was transferred in a 2 mL tube and extraction continued following manufacturer's instructions to produce one eDNA sample per pond.

TARGETED qPCR

Prior to testing for *T. cristatus*, all extracted samples were tested for PCR inhibitors and sample degradation using methodology outlined by Biggs *et al.* (2015), where an Internal Positive Control was included in qPCR reactions of eDNA samples and a sample considered inhibited if replicates showed different C_q values (where samples move into the exponential phase of qPCR amplification). Targeted qPCR was carried out as part of the *T. cristatus* monitoring programmes mentioned above in the laboratories at Fera and ADAS during 2015 using a standardised protocol (Biggs *et al.* 2015). Extracted DNA was amplified by TaqMan probe qPCR using published primers and probe to amplify an 81 bp fragment of the cytochrome b gene: TCCBL (5'-CGTAAACTACGGCTGACTAGTACGAA-3'), TCCBR (5-CCGATGTGTATGTAGATGCAAACA) and TCCB_Probe (5'-CCACGCTAACGGAGCCTCGC-3') (Thomsen *et al.* 2012). PCR reactions were set up in a total volume of 25 µL consisting of: 3 µL of extracted template DNA, 1 µL of each primer (0.4 µM), 1 µL of probe (0.1 µM), 1x TaqMan® Environmental Master Mix 2.0 (containing AmpliTaq GOLD DNA polymerase, Life Technologies) and ddH_2O. The PCR included an initial incubation for 5 min at 50 °C, then a 10 min denaturation step at 95 °C, followed by 55 cycles of denaturation at 95 °C for 30 s and annealing at 56.3 °C for 1 min. For each sample, 12 qPCR replicates were performed and a sample recorded as positive for *T. cristatus* if one or more qPCR replicates were positive. Positive (*T. cristatus* DNA: 1×10^{-1} ng/µL to 1×10^{-4} ng/µL) and negative controls (ddH_2O) were also included on each plate in quadruplicate. Following qPCR, the eDNA samples were placed in storage at -80 ^{o}C.

Appendix 2: Reference database construction

A custom, phylogenetically curated reference database of the target region was created for UK vertebrate species. For freshwater fish, we used a previously created database comprising 67 fish species, which includes all known native and non-native species in the UK and our positive control *Rhamphochromis esox*, a species of cichlid from Lake Malawi (Hänfling *et al.* 2016). For all remaining vertebrate species recorded in the UK (Natural History Museum UK Species Database, 2016), custom, phylogenetically curated reference databases were constructed using the ReproPhylo environment (Szitenberg *et al.* 2015) in a Jupyter notebook (Jupyter Team 2016). Each vertebrate group was processed separately in order to build Muscle alignments (Edgar 2004) and visualise constructed phylogenetic trees. Species lists containing the binomial nomenclature of UK vertebrate species were constructed using the Natural History Museum UK Species Database. All vertebrates recorded in the UK were included. The BioPython script performed a GenBank search based on the species lists and downloaded all available mitochondrial 12S ribosomal RNA (rRNA) sequences for specified species. Proportion of reference sequences available for species varied within each vertebrate group: amphibians 100.00% (N = 21), reptiles 90.00% (N = 20), birds 55.88% (N = 621), and mammals (83.93%, N = 112), Where there were no records on GenBank for a UK species, the database was supplemented with downloaded sequences belonging to sister species in the same genus. Species that had no 12S rRNA records on Genbank are provided in Table S1.

Redundant sequences were removed by clustering at 100% similarity using vsearch 1.1 (https://github.com/torognes/vsearch). Due to high proportion of partial 12S rRNA records on GenBank for the majority of UK species, only sequences longer than 500 bp were processed initially to increase alignment robustness to large gaps. Short sequences can cause problems in global paired alignments where the alignment algorithm attempts to align them to longer sequences. Short 12S rRNA sequences (<500 bp) were later incorporated into the existing long 12S rRNA alignment using the hmmer v3 program suite (HMMER development team 2016) to construct a Hidden Markov Model alignment containing sequences of all lengths. Alignments were trimmed using trimAl (Capella-Gutiérrez, Silla-Martínez & Gabaldón 2009). Maximum likelihood trees were inferred with RAxML 8.0.2 (Stamatakis 2006) using the GTR+gamma model of substitutions. The complete alignments were then processed using SATIVA (Kozlov *et al.* 2016) for automated identification of 'mislabelled' sequences which could cause conflict in downstream analyses. Putatively mislabelled sequences were removed and process of alignment and phylogenetic tree construction repeated for manual investigation of sequences. The resultant databases (i.e. curated non-redundant reference databases) contained: 198 amphibian sequences from 20/21 species, 112 reptile sequences from 19/20 species, 272 fish sequences from 60/62 species, 940 mammal sequences from 95/112 species, and 622 bird sequences from 347/621 species. Databases for each vertebrate group were concatenated and the combined vertebrate database used for *in silico* validation of primers.

The amphibian database was supplemented by Sanger sequences obtained from tissue of

great crested newt *Triturus cristatus*, smooth newt *Lissotriton vulgaris*, Alpine newt *Mesotriton alpestris*, common toad *Bufo bufo* (supplied by DICE, University of Kent, under licence from Natural England) and common frog *Rana temporaria* (supplied by University of Glasgow). Amphibian DNA was extracted from tissue samples using a Qiagen® DNeasy Blood & Tissue kit under licence from Natural England by H. Rees. Reference sequences of the entire 12S rRNA region were generated by three sets of novel primers:

**GCN (61 °C)**:	Newt_F1	5'-GCACTGAAAATGCTAAGACAGA-3'
	Newt_R6	5'-CAGGTATTTTCTCGGTGTAAGCA-3'
**Newts (59 °C):**	Newt_F2	5'-GCACTGAAAATGCTAAGACAG-3'
	Newt_R1	5'-TCTCGGTGTAAGCAAGATGC-3'
**Anura (57 °C):**	AnuraShort_F2	5'-TCCACTGGTCTTAGGAGCCA-3'
	AnuraShort_R1	5'-ACCATGTTACGACTTGCCTC-3'

Primers were designed from an alignment of tRNA, 12S and 16S rRNA regions in UK Caudata and Anura species. PCR reactions were performed in 25 µL volumes containing: 12.5 µL of Bioline® MyTaq™ Red Mix, 1 µL of forward and reverse primer (final concentration - 0.04 µM), 8.5µL of molecular grade sterile water (Fisher Scientific) and 2 µL DNA template. PCRs were performed on an Applied Biosystems® Veriti Thermal Cycler with the following profile: 95 °C for 3 min, 35 cycles of 95 °C for 30 sec, ~ °C for 60 sec and 72 °C for 90 sec, followed by a final elongation step at 72 °C for 10 min. Purified PCR products were Sanger sequenced directly (Macrogen Europe) in both directions using the PCR primers. Sequences were edited using CodonCode Aligner (CodonCode Corporation, Centerville, MA, USA).

The complete reference database compiled in GenBank format has been deposited in the dedicated GitHub repository for this study (https://github.com/HullUni-bioinformatics/Harper_et_al_2018) which has been permanently archived (https://doi.org/10.5281/zenodo.1188709).

Table S1. List of species for which no 12S rRNA records were available on Genbank. Only UK species which had no records for sister species within the same genus are included.

**Common name**	**Binomial nomenclature**
North Atlantic right whale	*Eubalaena glacialis*
Common kingfisher	*Alcedo atthis*
Trumpeter finch	*Bucanetes githagineus*
Green heron	*Butorides virescens*
Greater short-toed lark	*Calandrella brachydactyla*
Lesser short-toed lark	*Calandrella rufescens*
Lapland longspur	*Calcarius lapponicus*
Wilson's warbler	*Cardellina pusilla*
Rufuous-tailed scrub robin	*Cercotrichas galactotes*
MacQueen's bustard	*Chlamydotis macqueenii*
Lark sparrow	*Chondestes grammacus*
White-throated dipper	*Cinclus cinclus*
Great spotted cuckoo	*Clamator glandarius*
Long-tailed duck	*Clangula hyemalis*
Corn crake	*Crex crex*
Crested lark	*Galerida cristata*
European storm petrel	*Hydrobates pelagicus*
Little gull	*Hydrocoloeus minutus*
White-throated robin	*Irania gutturalis*
Hooded merganser	*Lophodytes cucullatus*
European crested tit	*Lophophanes cristatus*
Woodlark	*Lullula arborea*
Siberian blue robin	*Larvivora cyane*
Rufous-tailed robin	*Larvivora sibilans*
Thrush nightingale	*Luscinia luscinia*
Common nightingale	*Luscinia megarhynchos*
Bluethroat	*Luscinia svecica*
Black scoter	*Melanitta americana*
Velvet scoter	*Melanitta fusca*
Common scoter	*Melanitta nigra*

Surf scoter	*Melanitta perspicillata*
Bimaculated lark	*Melanocorypha bimaculata*
Calandra lark	*Melanocorypha calandra*
White-winged lark	*Melanocorypha leucoptera*
Black lark	*Melanocorypha yeltoniensis*
Song sparrow	*Melospiza melodia*
Black-and-white warbler	*Mniotilta varia*
Common rock thrush	*Monticola saxatilis*
Blue rock thrush	*Monticola solitarius*
Wilson’s storm petrel	*Oceanites oceanicus*
Band-rumped storm petrel	*Oceanodroma castro*
Leach’s storm petrel	*Oceanodroma leucorhoa*
Swinhoe’s storm petrel	*Oceanodroma monorhis*
Tennessee warbler	*Oreothlypis peregrina*
Northern waterthrush	*Parkesia noveboracensis*
Savannah sparrow	*Passerculus sandwichensis*
Rosy starling	*Pastor roseus*
American cliff swallow	*Petrochelidon pyrrhonota*
Steller’s eider	*Polysticta stelleri*
Eurasian crag martin	*Ptyonoprogne rupestris*
Sand martin	*Riparia riparia*
Whinchat	*Saxicola rubetra*
African stonechat	*Saxicola torquatus*
Northern parula	*Setophaga americana*
Hooded warbler	*Setophaga citrina*
American yellow warbler	*Setophaga petechia*
American redstart	*Setophaga ruticilla*
Wallcreeper	*Tichodroma muraria*
Brown thrasher	*Toxostoma rufum*
Golden-winged warbler	*Vermivora chrysoptera*

Appendix 3: Vertebrate eDNA metabarcoding workflow

PRIMER VALIDATION

Vertebrate DNA from eDNA samples was amplified with published 12S rRNA primers 12S-V5-F (5'-ACTGGGATTAGATACCCC-3') and 12S-V5-R (5'-TAGAACAGGCTCCTCTAG-3') (Riaz *et al.* 2011). Primers were validated for the present study *in silico* using ecoPCR software (Ficetola *et al.* 2010; Bellemain *et al.* 2010) against a custom, phylogenetically curated reference database for UK vertebrates. Parameters were set to allow a fragment size of 50-250 bp and maximum of three mismatches between the primer pair and each sequence in the reference database. Primers were previously validated *in vitro* for UK fish communities by Hänfling *et al.* (2016) and here were also validated against tissue DNA extracted from UK amphibian species (supplied by DICE, University of Kent, and University of Glasgow): great crested newt, smooth newt, palmate newt *Lissotrition helveticus*, Alpine newt, common frog and common toad. Primer validation tests were performed at University of Hull in a separate laboratory situated on a different floor to the dedicated eDNA laboratory. A dilution series (10^0 to 10^{-8}) was performed for DNA (standardised to 5 ng/µL) from each species to identify the limit of detection (LOD) for each species. Molecular grade sterile water (Fisher Scientific) substituted template DNA for the PCR negative control.

TWO-STEP PCR PROTOCOL

A two-step PCR protocol was performed on eDNA samples at University of Hull. Dedicated rooms were available for pre-PCR and post-PCR processes. Pre-PCR processes were performed in a dedicated eDNA laboratory, with separate rooms for filtration, DNA extraction and PCR preparation of sensitive environmental samples. PCR reactions were set up in a UV and bleach sterilized laminar flow hood. Eight-strip PCR tubes with individually attached lids were used instead of 96-well plates to minimise cross-contamination risk between samples (Port *et al.* 2016). After the first sequencing run revealed substantial human contamination across samples and PCR controls, reactions prepared for the second sequencing run were sealed with mineral oil as an additional measure against PCR contamination. For the first PCR, three replicates were performed for each sample to combat PCR stochasticity. Alternating PCR positive and negative controls were included on each PCR strip (six positive and negative controls on each 96-well plate), to screen for sources of potential contamination. The DNA used for the PCR positive control was *R. esox*, as occurrence in UK ponds is extremely rare or non-existent. The negative control substituted molecular grade sterile water for template DNA.

During the first PCR, the target region was amplified using the primers described above, including adapters (Illumina 2011). First step PCR reactions were performed in a final volume of 21.1 µL, using 2 µL of DNA extract as a template. The amplification mixture contained 10.5 µL of Bioline® MyTaq™ HS Red Mix, 1.05 µL of forward and reverse primer (final concentration -

0.5 μM) and 6.5 μL of molecular grade sterile water (Fisher Scientific). PCR was performed on an Applied Biosystems® Veriti Thermal Cycler and PCR conditions for the first component of the two-step protocol consisted of: an incubation step at 98 °C for 5 min, followed by 35 cycles of denaturation at 98 °C for 15 s, annealing at 56 °C for 20 s, and extension at 72 °C for 30 s with final extension at 72 °C for 10 min. PCR products were stored at 4 °C until fragment size was verified by visualising 5 μL of selected PCR products on 2% agarose gels (100 mL 0.5x TBE buffer, 2 g agarose powder). Gels were then stained with ethidium bromide and imaged using Image Lab Software. A PCR product was deemed positive where there was an amplification band on the gel that was of the expected size (200-300 bp). PCR replicates for each sample were pooled in preparation for the addition of Illumina indexes in the second PCR, which resulted in 63.3 μL of PCR product for each sample. PCR positive and negative controls were not pooled to allow individual purification and sequencing of all 228 PCR controls. All PCR products (30 μL samples and 15 μL PCR controls) were then purified to remove excess primer using E.Z.N.A. Cycle Pure V-Spin Clean-Up Kits (VWR International) following manufacturer centrifugation protocol. Eluted DNA was stored at -20 °C until the second PCR could be performed.

In the second PCR, Molecular Identification (MID) tags (unique 8-nucleotide sequences) and Illumina MiSeq adapter sequences were bound to the amplified product. These tags were included in the forward and reverse primers resulting in indexed primers for second PCR (O'Donnell *et al.* 2016). For each second PCR plate, 96 unique tag combinations were created by combining eight unique forward tags with 12 unique reverse tags or vice versa (Kitson *et al.* 2018). A total of 384 unique tag combinations were achieved, allowing samples to be distinguished during bioinformatics analysis. Second step PCR reactions were performed in eight-strip PCR tubes with individually attached lids in a final volume of 21.1 μL, using 2 μL of purified DNA from the first PCR product as a template. The amplification mixture contained 10.5 μL of Bioline® MyTaq™ HS Red Mix, 2.1 μL of tagged primer mix (final concentration - 0.5 μM) and 6.5 μL of molecular grade sterile water (Fisher Scientific). PCR was performed on an Applied Biosystems® Veriti Thermal Cycler with the following profile: denaturation at 95 °C for 3 min, followed by 12 cycles of annealing at 98 °C for 20 s and extension at 72 °C for 30 s with final extension at 72 °C for 5 min. PCR products were stored at 4 °C before they were all visualised on 2% agarose gels (100 mL 0.5x TBE buffer, 2 g agarose powder) using 5 μL PCR product. Gels were then stained with ethidium bromide and imaged using Image Lab Software. Again, PCR products were deemed positive where there was an amplification band on the gel that was of the expected size (200-300 bp). Amplification bands were found to be present in some of the negative controls thus all negative controls were included for sequencing.

LIBRARY PREPARATION

All remaining library preparation was conducted at Fera. PCR products were transferred to a new 96-well PCR plate for individual purification with AMPure® XP beads and a magnetic stand. The Illumina PCR clean-up protocol was adapted to use 18.6 μL AMPure® XP beads (1.2x PCR product) to 15-16 μL PCR product. Illumina protocol was then followed until the beads were

resuspended in 15 µL molecular grade water and incubated at room temperature for 5 minutes. The supernatant without beads in each well were not transferred to a new plate due to low volumes of purified product. Further pipetting may have resulted in loss of DNA. Each plate was sealed and stored at 4 °C until quality assurance. A Quant-IT™ PicoGreen™ dsDNA Assay was conducted for all samples on a ThermoFisher 96-well microplate reader. Samples were then normalised and pooled to create 4 nM pooled libraries before quantification using a Qubit™ dsDNA HS Assay. Both libraries passed quality assurance with concentrations of 2.62 ng/µl and 4.14 ng/µl respectively. A Tapestation D1000K assay was then used to check and compare size of the pooled libraries to selected samples. The pooled libraries were 272 bp and 299 bp (expected 286 bp) with samples in the same range. Equimolar libraries (4 nM) were then created using tapestation trace size estimates and Qubit concentrations. Libraries were run at 12 pM concentration on an Illumina MiSeq using 2 x 300 bp V3 chemistry. Both libraries included a 10% PhiX DNA spike-in control to improve clustering during initial sequencing.

BIOINFORMATIC PROCESSING

Raw reads were quality trimmed using Trimmomatic v0.32 (Bolger, Lohse & Usadel 2014), both from the read ends (minimum per base phred score Q30), as well as across sliding windows (window size 5bp; minimum average phred score Q30). Reads were clipped to a maximum length of 110 bp and reads shorter than 90 bp after quality trimming were discarded. To reliably exclude adapters and PCR primers, the first 25 bp of all remaining reads were also removed. Sequence pairs were merged into single high quality reads using FLASH v1.2.11 (Magoč & Salzberg 2011), if a minimum of 10 bp overlap with a maximum of 10% mismatch was detected between pairs. For reads that were not successfully merged, only forward reads were kept. To reflect our expectations with respect to fragment size, a final length filter was applied and only sequences of length 80-120 bp were retained. These were screened for chimeric sequences against our custom reference database using the uchime algorithm (Edgar *et al.* 2011), as implemented in vsearch v1.1 (Rognes *et al.* 2016). Redundant sequences were removed by clustering at 97% identity ('--cluster_fast' option) in vsearch v1.1 (Rognes *et al.* 2016). Clusters represented by less than five sequences were considered sequencing error and omitted from further analyses. Non-redundant sets of query sequences were then compared against our custom reference database using BLAST (Zhang *et al.* 2000). For any query matching with at least 98% identity to a reference sequence across more than 80% of its length, putative taxonomic identity was assigned using a lowest common ancestor (LCA) approach based on the top 10% BLAST matches. Sequences that could not be assigned (non-target sequences) were subjected to a separate BLAST search against the complete NCBI nucleotide (nt) database at 98% identity to determine the source via LCA as described above.

Appendix 4: Data analysis

MANIPULATION OF METABEAT DATASET

Non-target sequence assignments and original assignments at 98% identity were merged. Any spurious assignments (i.e. non-UK species, invertebrates and bacteria) were removed from the dataset. Assignments to genera or families which contained only a single UK representative were manually assigned to that species. In our dataset, only genus *Strix* was reassigned to tawny owl *Strix aluco*. Where family and genera assignments containing a single UK representative did have reads assigned to species, reads from all assignment levels were merged and manually assigned to that species. Consequently, all taxonomic assignments included in the final database were of species resolution. A total of 60 species were detected by eDNA metabarcoding. Mis-assignments in our dataset were then corrected; again, only one instance was identified. Scottish wildcat *Felis silvestris* was reassigned to domestic cat *Felis catus* on the basis that Scottish wildcat does not occur where ponds were sampled (Kent, Lincolnshire and Cheshire).

GLMM COMPARISON OF eDNA METHODS FOR *T. CRISTATUS* DETECTION

Initially, a Poisson distribution was specified but tests using the R package RVAideMemoire v 0.9-45-2 (Hervé 2015) revealed models with this distribution were overdispersed. Models with a quasi-Poisson and zero-inflated distribution failed to resolve overdispersion (Ver Hoef & Boveng 2007). A negative binomial distribution was used to control for aggregation in the count data and prevent biased parameter estimates (Harrison 2014). Model overdispersion remained unresolved but model fit was improved. Model fit was assessed using the Hosmer and Lemeshow Goodness of Fit Test (Hosmer & Lemeshow 2000) within the R package 'ResourceSelection' v0.2-4 (Lele *et al.* 2016). Model predictions were obtained using the predictSE() function in the 'AICcmodavg' package v2.0-3 (Mazerolle 2017) and upper and lower 95% CIs were calculated from the standard error of the predictions.

Table S2. List of species detected in PCR positive controls by eDNA metabarcoding and corresponding species-specific false positive sequence threshold applied.

**Common name**	**Binomial name**	**False positive sequence threshold**
European eel	*Anguilla anguilla*	0.000094
Common carp	*Cyprinus carpio*	0.000163
Common minnow	*Phoxinus phoxinus*	0.001287
Common roach	*Rutilus rutilus*	0.000291
European chub	*Squalius cephalus*	0.004080
Three-spined stickleback	*Gasterosteus aculeatus*	0.066667
Atlantic herring	*Clupea harengus*	0.000115
Common toad	*Bufo bufo*	0.066667
Common frog	*Rana temporaria*	0.000596
Smooth newt	*Lissotriton vulgaris*	0.066667
Great crested newt	*Triturus cristatus*	0.000276
Green-winged teal	*Anas carolinensis*	0.000322
Eurasian coot	*Fulica atra*	0.000223
Common moorhen	*Gallinula chloropus*	0.000179
Common starling	*Sturnus vulgaris*	0.000139
Human	*Homo sapiens*	0.253333
Brown rat	*Rattus norvegicus*	0.000467
Cow	*Bos taurus*	0.003542
Pig	*Sus scrofa*	0.000877

Appendix 5: Additional results

IN SILICO PRIMER VALIDATION

The *in silico* analysis confirmed high taxonomic coverage (59.0% of target vertebrate species amplified) and resolution of the 12S rRNA primers. A wide range of UK vertebrate taxa were amplified, with fragment length ranging from 90-114 bp. The primers amplified 16/21 amphibian species, including great crested newt. Palmate newt, Italian crested newt *Triturus carnifex*, brown cave salamander *Hydromantes genei*, marsh frog *Pelophylax esculentus* and agile frog *Rana dalmatina* were not amplified *in silico*. All sequences from these species were manually aligned to the primers using the alignment viewer and editor AliView (Larsson 2014), confirming potential for amplification. The primers amplified 47/67 fish species, including the threatened European eel *Anguilla anguilla*, but amplification of UK freshwater fish assemblages was confirmed *in vitro* by Hänfling *et al.* (2016). The primers amplified 14/20 reptile species including slow worm *Anguis fragilis* and common lizard *Zootoca vivipara*. Reference sequences were not available for one species and a further five species were not amplified. Primers were only validated for 282/621 bird species (including common waterfowl species). There were no 12S rRNA data available for 243/621 bird species and a further 96 species were not amplified. Similarly, no reference data were available for nine mammal species (bats and marine mammals) and a further 15 species were not amplified. Only 88/112 mammal species were validated. Several marine mammal species were not amplified but would not be found in freshwater ponds. However, priority species for freshwater management, such as water vole *Arvicola amphibius* and American mink *Mustela vison*, were not amplified alongside other species of bat, vole and shrew that may frequent ponds.

IN VITRO PRIMER VALIDATION

Bands were observed by agarose gel electrophoresis for all amphibian tissue tested, including palmate newt which was not amplified *in silico*, and no bands were observed in NTCs. The LOD was variable for each species: great crested newt, palmate newt, common frog and common toad were not amplified below 5×10^{-4} ng/µl, whereas Alpine newt was not amplified below 5×10^{-3} ng/µl and smooth newt below 5×10^{-5} ng/µl. Due to sheer number of and legislation surrounding many UK amphibian, reptile, bird and mammal species, conducting *in vitro* testing for all target taxa was unfeasible and metabarcoding proceeded on the basis of *in silico* amplification.

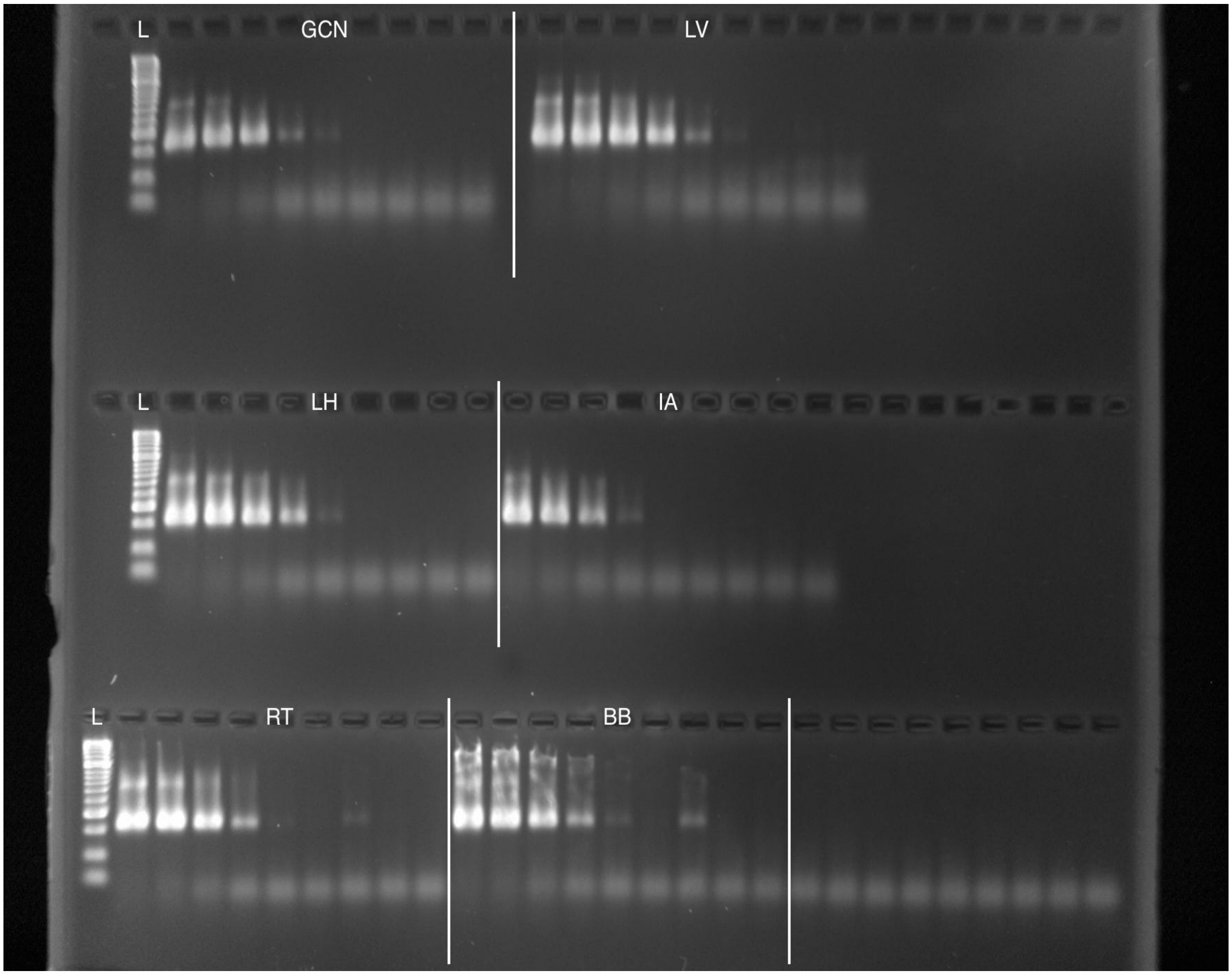

Figure S1. Gel image showing results of *in vitro* primer validation for each species: great crested newt (GCN), smooth newt (LV), palmate newt (LH), Alpine newt (IA), common frog (RT) and common toad (BB).

VERTEBRATE METABARCODING

Table S3. Summary statistics for each Illumina MiSeq run.

MiSeq Run	Date	Samples	Controls	Raw reads	Reads passing QC	Non-redundant reads	Reads taxonomically assigned	Unassigned reads
1	01/04/16	266	114	36,236,862	26,294,906	14,141,237	13,126,148	1,015,089
2	29/04/16	266	114	32,900,914	26,451,564	14,081,788	13,113,143	968,976

Table S4. Summary of read counts and the overall proportion of reads assigned to taxonomic levels for each Illumina MiSeq run.

MiSeq Run	Species	Genus	Family	Order	Class	Overall assignment (%)
1	10,185,014	1,438,216	963,865	12,454	526,599	92.82
2	9,419,096	1,237,427	1,899,932	10,723	545,965	93.12

Table S5. Sequence read count data obtained for 12S rRNA gene made available as an excel spreadsheet.

Table S6. Summary of species detected by eDNA metabarcoding of freshwater pond samples (N = 532).

Common name	Binomial name	No. samples detected
European eel	*Anguilla anguilla*	15
Common barbel	*Barbus barbus*	2
Crucian carp	*Carassius carassius*	2
Common carp	*Cyprinus carpio*	41
Common minnow	*Phoxinus phoxinus*	13
Common roach	*Rutilus rutilus*	72
European chub	*Squalius cephalus*	21
Stone loach	*Barbatula barbatula*	15
Northern pike	*Esox lucius*	17
European bullhead	*Cottus gobio*	14
Three-spined stickleback	*Gasterosteus aculeatus*	56
Ninespine stickleback	*Pungitius pungitius*	15
Ruffe	*Gymnocephalus cernua*	1
Rainbow trout	*Oncorhynchus mykiss*	3
Common toad	*Bufo bufo*	42
Marsh frog	*Pelophylax ridibundus*	1
Common frog	*Rana temporaria*	120
Palmate newt	*Lissotrition helveticus*	5
Smooth newt	*Lissotriton vulgaris*	152
Great crested newt	*Triturus cristatus*	149
Green-winged teal	*Anas carolinensis*	7
Eurasian oystercatcher	*Haematopus ostralegus*	1
Common buzzard	*Buteo buteo*	4
Common pheasant	*Phasianus colchicus*	25
Domesticated turkey	*Meleagris gallopavo*	11
Helmeted guineafowl	*Numida meleagris*	1
Eurasian coot	*Fulica atra*	48
Common moorhen	*Gallinula chloropus*	215
Eurasian jay	*Garrulus glandarius*	7

European goldfinch	*Carduelis carduelis*	1
Dunnock	*Prunella modularis*	4
Eurasian nuthatch	*Sitta europaea*	1
Common starling	*Sturnus vulgaris*	4
Melodius warbler	*Hippolais polyglotta*	2
Grey heron	*Ardea cinerea*	1
Great spotted woodpecker	*Dendrocopus major*	1
Green woodpecker	*Picus viridis*	2
Tawny owl	*Strix aluco*	1
Dog	*Canis lupus*	65
Red fox	*Vulpes vulpes*	9
Eurasian otter	*Lutra lutra*	1
European badger	*Meles meles*	7
European polecat	*Mustela putorius*	1
Common pipistrelle	*Pipistrellus pipistrellus*	1
Eurasian water shrew	*Neomys fodiens*	9
Common shrew	*Sorex araneus*	1
European hare	*Lepus europaeus*	1
European rabbit	*Oryctolagus cuniculus*	24
Horse	*Equus caballus*	3
European water vole	*Arvicola amphibius*	16
Bank vole	*Myodes glareolus*	9
House mouse	*Mus musculus*	16
Brown rat	*Rattus norvegicus*	39
Grey squirrel	*Sciurus carolinensis*	57
Cow	*Bos taurus*	179
Sheep	*Ovis aries*	42
Red deer	*Cervus elaphus*	2
Reeve's muntjac	*Muntiacus reevesi*	3
Pig	*Sus scrofa*	140
Cat	*Felis catus*	16

Table S7. Summary of contaminants detected in PCR negative, or No Template Controls (NTCs), that occurred at high proportion of the total read count (> 1%). Maximum frequency and read count across all NTCs are provided for each contaminant.

**Common name**	**Binomial name**	**No. NTCs detected**	**Max. proportion**	**Max. read count**
Great crested newt	*Triturus cristatus*	6	93.0%	307
Smooth newt	*Lissotriton vulgaris*	12	100.0%	55
Common frog	*Rana temporaria*	10	63.2%	13,120
Common toad	*Bufo bufo*	1	22.8%	46
Common roach	*Rutilus rutilus*	6	81.3%	25,441
European bullhead	*Cottus gobio*	4	91.4%	10,827
Three-spined stickleback	*Gasterosteus aculeatus*	2	25.6%	166
Stone loach	*Barbatula barbatula*	1	6.2%	1,165
Common moorhen	*Gallinula chloropus*	4	41.8%	140
Mouse	*Mus musculus*	2	96.1%	1,759
Dog	*Canis lupus*	1	2.8%	18
Pig	*Sus scrofa*	1	97.7%	14,622
Sheep	*Ovis aries*	1	30.6%	589

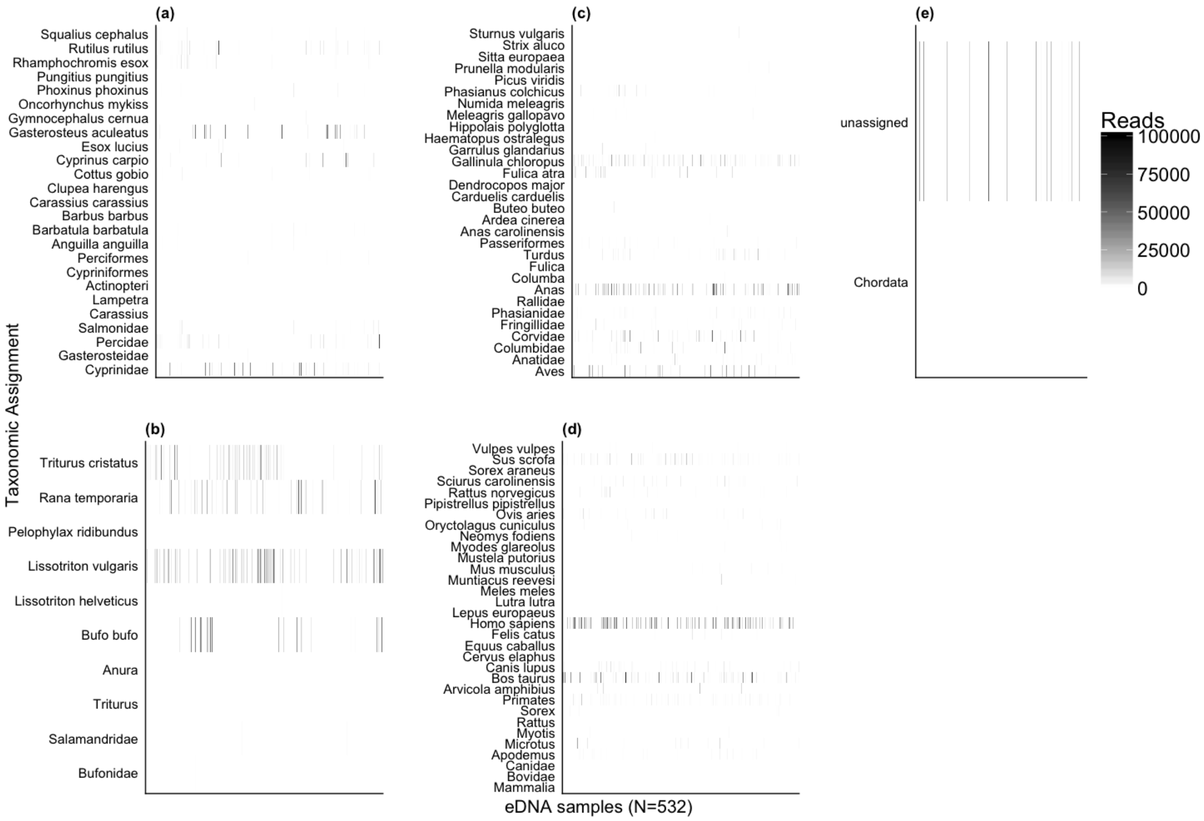

Figure S2. Heat maps of sequence read distribution for taxonomic assignments in each vertebrate group across all eDNA samples: **(a)** fish, **(b)** amphibians, **(c)** birds, **(d)** mammals and **(e)** other. Detections exceeding 100,000 reads (e.g. cow *Bos taurus*) were omitted during plotting to improve visualisation of lower read assignments in the dataset, but the data were not adjusted in this process. Each species was present in at least one sample although low read counts are not always visible.

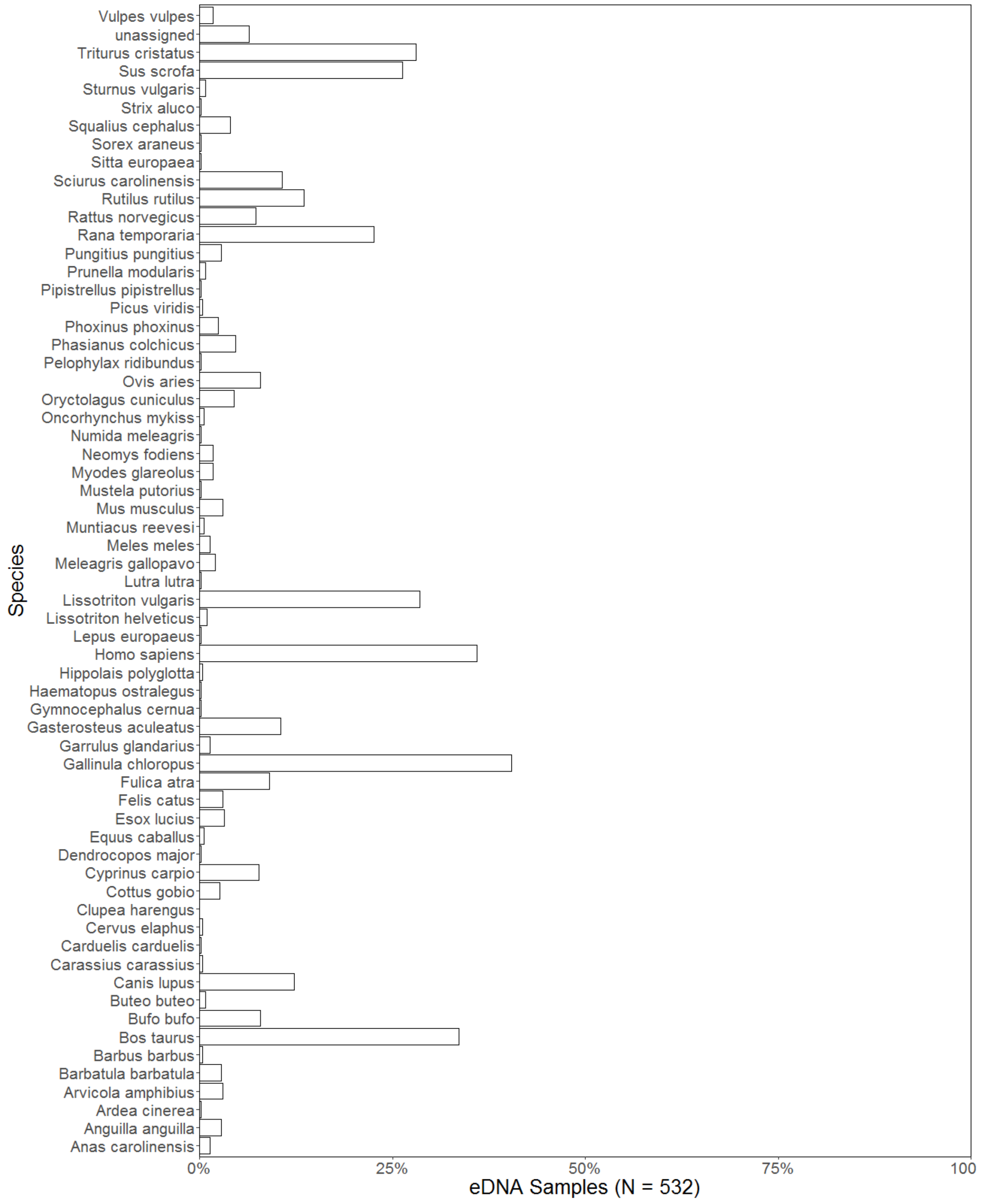

Figure S3. Proportion of eDNA samples in which each species was detected by eDNA metabarcoding.

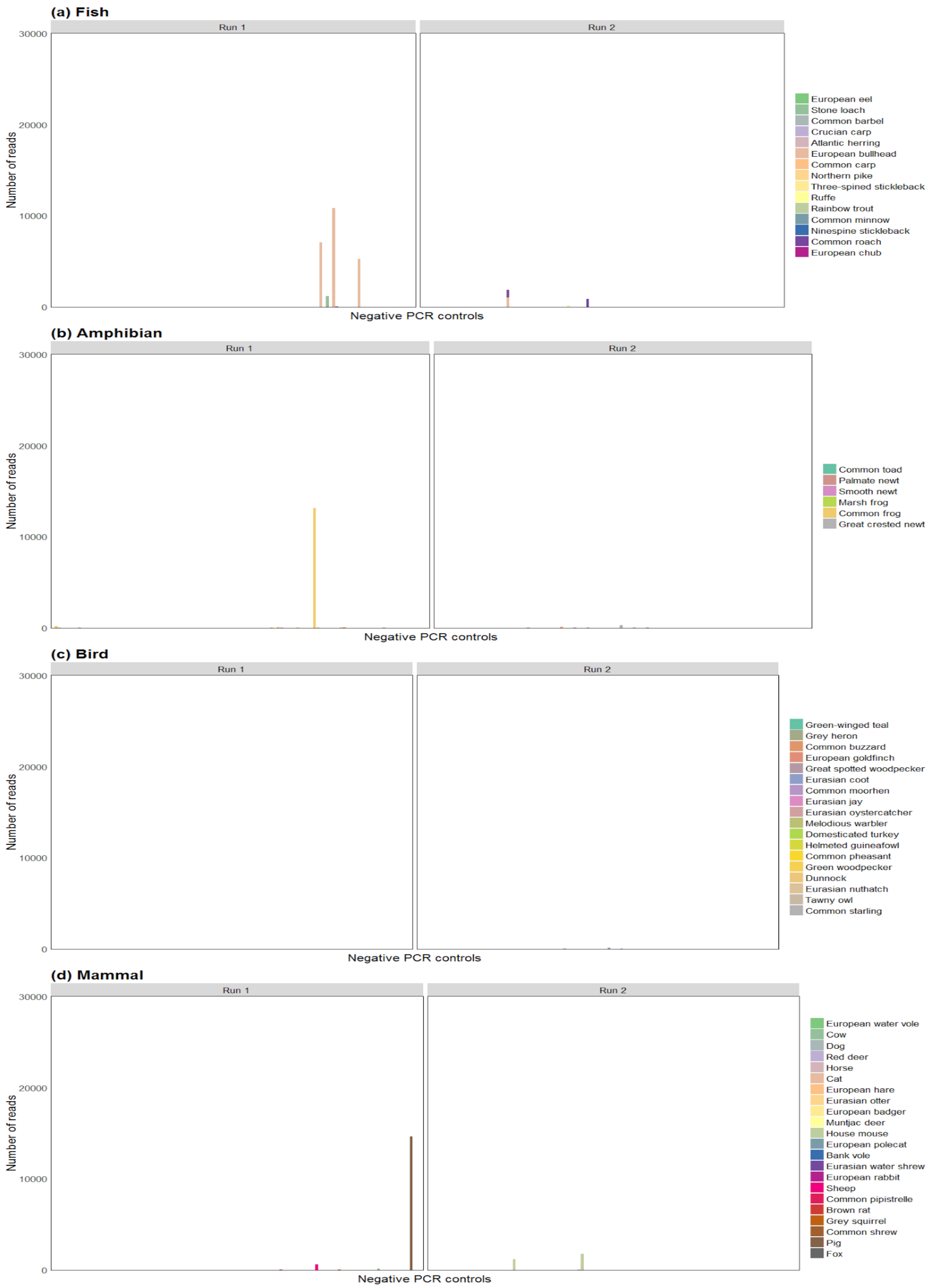

Figure S4. Presence of foreign DNA in PCR negative controls across sequencing runs. Highest contamination was observed from fish species, common roach *Rutilus rutilus* and European bullhead *Cottus gobio*, in addition to common frog *Rana temporaria* and pig *Sus scrofa*. Common roach occurred in six PCR negative controls, two of which exceeded 100 reads. European bullhead occurred in four PCR negative controls but all exceeded 1,000 reads. Notably, common frog occurred in 13 PCR negative controls but only two exceeded 100 reads, with 180 and 13,120 reads. Pig occurred in one PCR negative control only but exceeded 14,000 reads. Contamination from other species was relatively low with few species exceeding 100 sequence reads.

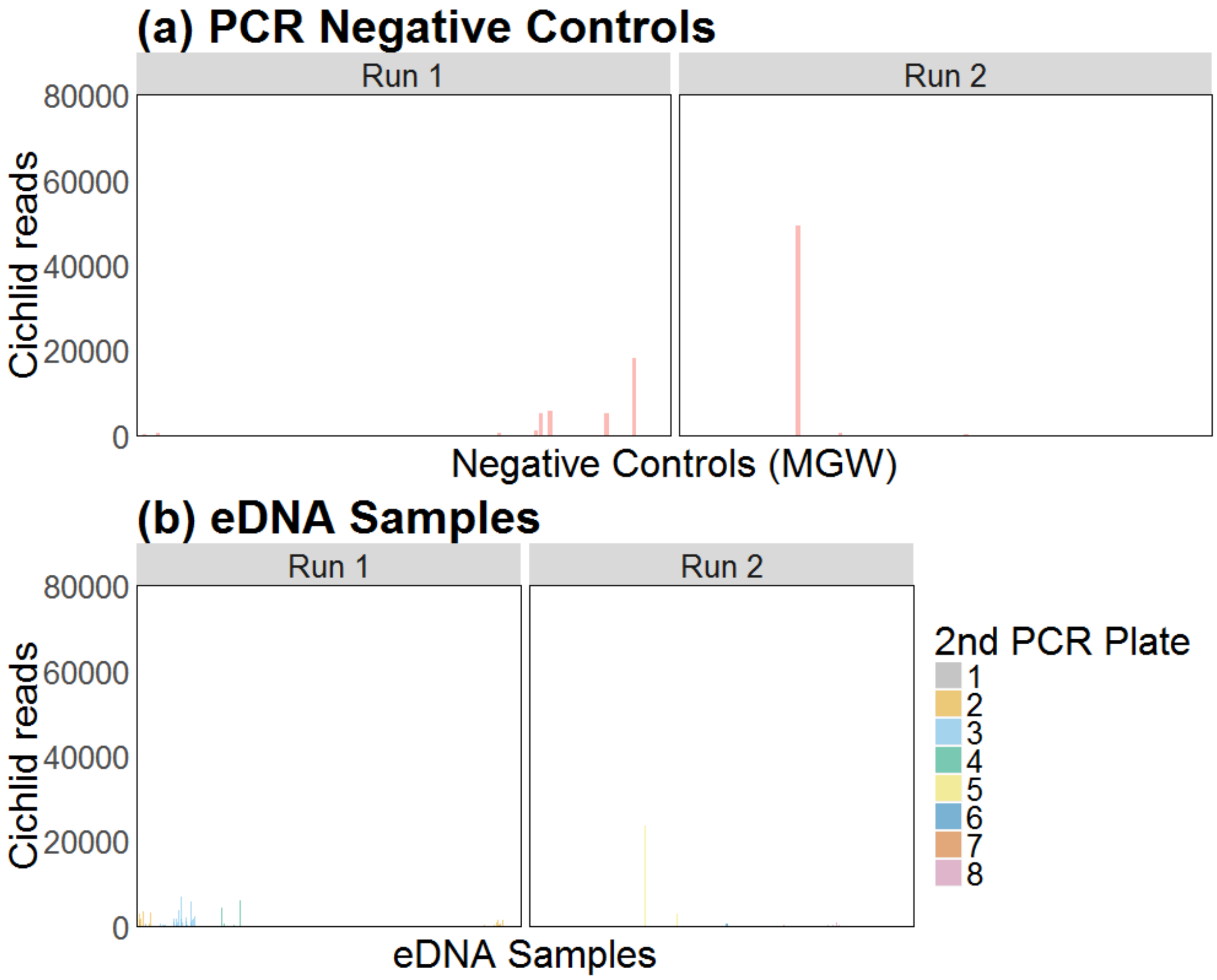

Figure S5. Presence of cichlid DNA (PCR positive control) amongst PCR negative controls and eDNA samples. Contamination of PCR negative controls was more frequent on the first sequencing run but greater where it occurred during the second sequencing run. Contamination of environmental samples was most common on plates 3 and 4, which were also sequenced on the first MiSeq run.

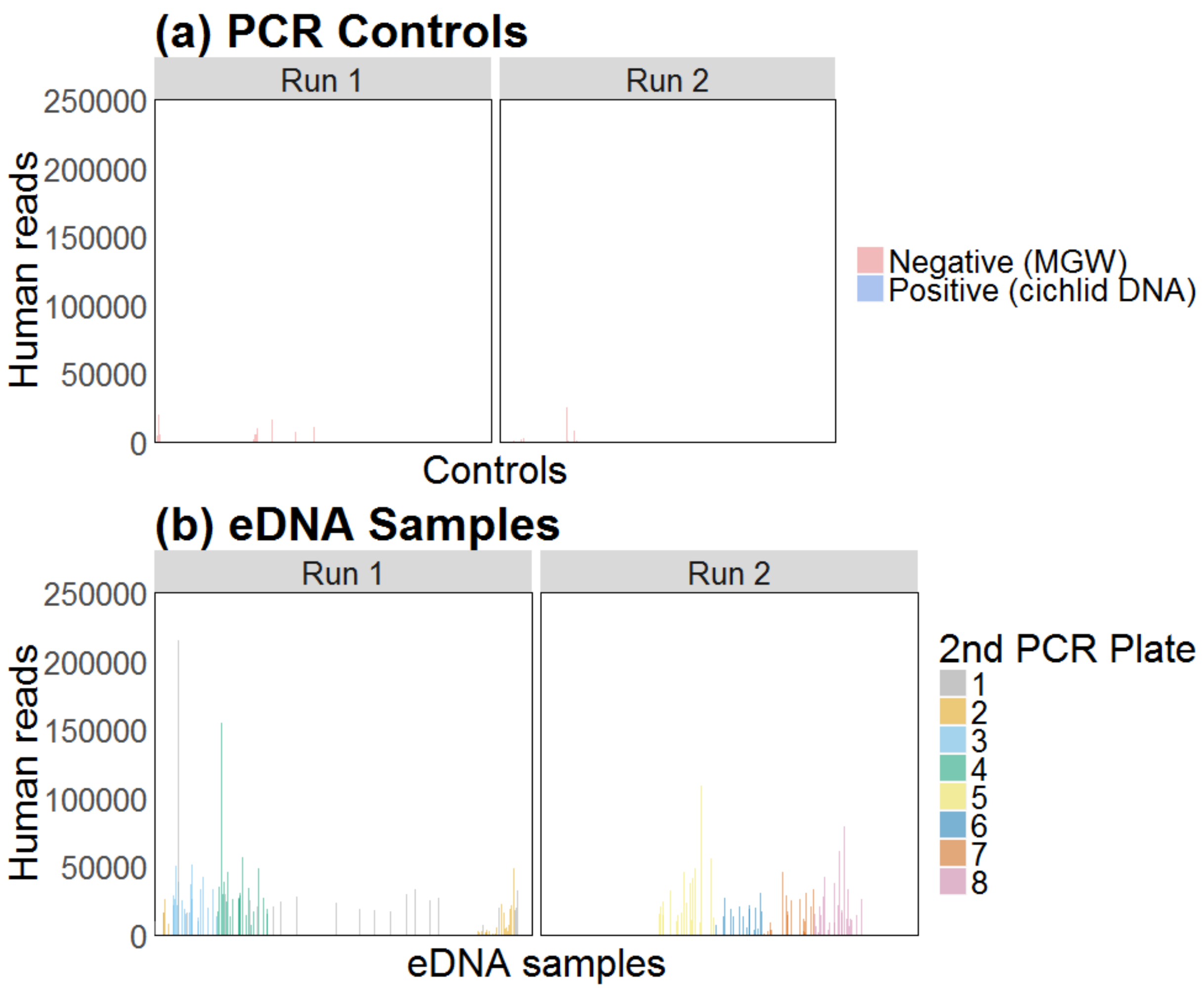

Figure S6. Presence of human DNA amongst PCR controls and eDNA samples. Contamination of PCR controls and environmental samples was less frequent in the second sequencing run. Human DNA contamination was most abundant in environmental samples on PCR plates 1, 3, 4 and 5.

eDNA METABARCODING VS qPCR FOR *T. CRISTATUS* DETECTION

Table S8. Summary of agreement (+) and disagreement (-) between egg searches, qPCR NT, qPCR TA, metabarcoding NT, and metabarcoding TA for *T. cristatus* detection in ponds (N = 532).

**Method**	**Egg search** +	**qPCR NT** +	**qPCR TA** +	**Metabarcoding NT** +	**Metabarcoding TA** +
**Egg search** -	*58 (+)* *448 (-)*	202	126	133	106
**qPCR NT** -	7	*265(+)* *267 (-)*	0	21	11
**qPCR TA** -	18	91	*174 (+)* *358 (-)*	48	26
**Metabarcoding NT** -	21	104	40	*182 (+)* *350 (-)*	0
**Metabarcoding TA** -	23	127	51	33	*149 (+)* *383 (-)*

COMPARISON OF METHOD COST AND INVESTIGATOR EFFORT

Table S9. Details of expenditure breakdown provided as an excel spreadsheet.

References in Supporting Information

Bellemain, E., Carlsen, T., Brochmann, C., Coissac, E., Taberlet, P. & Kauserud, H. (2010) ITS as an environmental DNA barcode for fungi: an *in silico* approach reveals potential PCR biases. *BMC Microbiology*, **10**, 1-9.

Biggs, J., Ewald, N., Valentini, A., Gaboriaud, C., Dejean, T., Griffiths, R.A., Foster, J., Wilkinson, J.W., Arnell, A., Brotherton, P., Williams, P. & Dunn, F. (2015) Using eDNA to develop a national citizen science-based monitoring programme for the great crested newt (*Triturus cristatus*). *Biological Conservation*, **183**, 19–28.

Capella-Gutiérrez, S., Silla-Martínez, J.M. & Gabaldón, T. (2009) trimAl: a tool for automated alignment trimming in large-scale phylogenetic analyses. *Bioinformatics*, **25**, 1972–1973.

Edgar, R.C. (2004) MUSCLE: multiple sequence alignment with high accuracy and high throughput. *Nucleic Acids Research*, **32**, 1792–1797.

Edgar, R.C., Haas, B.J., Clemente, J.C., Quince, C. & Knight, R. (2011) UCHIME improves sensitivity and speed of chimera detection. *Bioinformatics*, **27**, 2194–2200.

Ficetola, G.F., Coissac, E., Zundel, S., Riaz, T., Shehzad, W., Bessière, J., Taberlet, P. & Pompanon, F. (2010) An *In silico* approach for the evaluation of DNA barcodes. *BMC Genomics*, **11**, 434.

Hänfling, B., Lawson Handley, L., Read, D.S., Hahn, C., Li, J., Nichols, P., Blackman, R.C., Oliver, A. & Winfield, I.J. (2016) Environmental DNA metabarcoding of lake fish communities reflects long-term data from established survey methods. *Molecular Ecology*, **25**, 3101-3119.

Harrison, X.A. (2014) Using observation-level random effects to model overdispersion in count data in ecology and evolution. *PeerJ*, **2**, e616.

Hervé, M. (2015) RVAideMemoire: Diverse basic statistical and graphical functions. R package version 0.9-36.

Hosmer, D.W. & Lemeshow, S. (2000) Multiple Logistic Regression. *Applied Logistic Regression*, pp. 31–46. John Wiley & Sons, Inc.

Illumina. 2011. Preparing 16S Ribosomal RNA Gene Amplicons for the Illumina MiSeq System. Illumina technical note.

Kitson, J.J.N., Hahn, C., Sands, R.J., Straw, N.A., Evans, D.M. & Lunt, D.H. (2018) Detecting host-parasitoid interactions in an invasive Lepidopteran using nested tagging DNA-metabarcoding. *Molecular ecology*.

Kozlov, A.M., Zhang, J., Yilmaz, P., Glöckner, F.O. & Stamatakis, A. (2016) Phylogeny-aware identification and correction of taxonomically mislabeled sequences. *Nucleic Acids Research*, **44**, 5022-5033.

Lele, S.R., Keim, J.L. & Solymos, P. (2016) ResourceSelection: Resource Selection (Probability) Functions for Use-Availability Data. R package version 0.3-2.

Magoč, T. & Salzberg, S.L. (2011) FLASH: fast length adjustment of short reads to improve genome assemblies. *Bioinformatics*, **27**, 2957–2963.

Magoč, A.M., Lohse, M. & Usadel, B. (2014) Trimmomatic: a flexible trimmer for Illumina sequence data. *Bioinformatics*, **30**, 2114–2120.

Mazerolle, M.J. (2017) AICcmodavg: Model selection and multimodel inference based on (Q)AIC(c). R package version 2.1-1.

Natural History Museum (2017) UK Species Database. Available at: http://www.nhm.ac.uk/our-science/data/uk-species/species/index.html [accessed 7th November 2017].

O'Donnell, J.L., Kelly, R.P., Lowell, N.C. & Port, J.A. (2016) Indexed PCR Primers Induce Template-Specific Bias in Large-Scale DNA Sequencing Studies. *PLoS One*, **11**, e0148698.

Port, J.A., O'Donnell, J.L., Romero-Maraccini, O.C., Leary, P.R., Litvin, S.Y., Nickols, K.J., Yamahara, K.M. & Kelly, R.P. (2016) Assessing vertebrate biodiversity in a kelp forest ecosystem using environmental DNA. *Molecular Ecology*, **25**, 527–541.

Riaz, T., Shehzad, W., Viari, A., Pompanon, F., Taberlet, P. & Coissac, E. (2011) ecoPrimers: inference of new DNA barcode markers from whole genome sequence analysis. *Nucleic Acids Research*, **39**, e145.

Rognes, T., Flouri, T., Nichols, B., Quince, C. & Mahé, F. (2016) VSEARCH: a versatile open source tool for metagenomics. *PeerJ*, **4**, e2584.

Stamatakis, A. (2006) RAxML-VI-HPC: maximum likelihood-based phylogenetic analyses with thousands of taxa and mixed models. *Bioinformatics*, **22**, 2688–2690.

Szitenberg, A., John, M., Blaxter, M.L. & Lunt, D.H. (2015) ReproPhylo: An Environment for Reproducible Phylogenomics. *PLoS Computational Biology*, **11**, e1004447.

Thomsen, P.F., Iversen, L.L., Wiuf, C., Rasmussen, M., Gilbert, M.T.P., Orlando, L. & Willerslev, E. (2012) Monitoring endangered freshwater biodiversity using environmental DNA. *Molecular Ecology*, **21**, 2565–2573.

Ver Hoef, J.M. & Boveng, P.L. (2007) QUASI-POISSON VS. NEGATIVE BINOMIAL REGRESSION: HOW SHOULD WE MODEL OVERDISPERSED COUNT DATA? *Ecology*, **88**, 2766-2772.

Zhang, Z., Schwartz, S., Wagner, L. & Miller, W. (2000) A greedy algorithm for aligning DNA sequences. *Journal of Computational Biology*, **7**, 203–214.
